# The impact of hyperlinks on reading text

**DOI:** 10.1371/journal.pone.0210900

**Published:** 2019-02-06

**Authors:** Gemma Fitzsimmons, Mark J. Weal, Denis Drieghe

**Affiliations:** 1 School of Psychology, University of Southampton, United Kingdom; 2 School of Electronics and Computer Science, University of Southampton, United Kingdom; University of Texas at El Paso, UNITED STATES

## Abstract

There has been debate about whether blue hyperlinks on the Web cause disruption to reading. A series of eye tracking experiments were conducted to explore if coloured words in black text had any impact on reading behaviour outside and inside a Web environment. Experiment 1 and 2 explored the saliency of coloured words embedded in single sentences and the impact on reading behaviour. In Experiment 3, the effects of coloured words/hyperlinks in passages of text in a Web-like environment was explored. Experiment 1 and 2 showed that multiple coloured words in text had no negative impact on reading behaviour. However, if the sentence featured only a single coloured word, a reduction in skipping rates was observed. This suggests that the visual saliency associated with a single coloured word may signal to the reader that the word is important, whereas this signalling is reduced when multiple words are coloured. In Experiment 3, when reading passages of text containing hyperlinks in a Web environment, participants showed a tendency to re-read sentences that contained hyperlinked, uncommon words compared to hyperlinked, common words. Hyperlinks highlight important information and suggest additional content, which for more difficult concepts, invites rereading of the preceding text.

## Introduction

One of the main differences between reading on and off the Web is that the materials that are being read on the Web contain hyperlinks embedded within the text. Two differences between reading hyperlinked words compared to plain words are explored in this study: Firstly, hyperlinks are typically coloured and therefore salient compared to the rest of the text and secondly, a hyperlink links one piece of information to another, perhaps on a separate page of the same website, or a different website all together. Hyperlinks are a tool to navigate the Web and the word chosen to be hyperlinked often represents the page the hyperlink is linking to. This paper systematically explores these features in order to understand the impact of hyperlinks on reading text on the Web.

Starting with saliency, hyperlinks are salient items that stand out from the rest of the text in some way. Visual saliency is a stimulus-driven signal that announces to us that a certain item or location is different to the rest of the visual field and is worthy of attention. For example, a lone red item in a field of green items will stand out to us and be salient compared to the rest of the items and draw our attention [1]. The way hyperlinks are denoted usually follows the convention that hyperlinks are denoted in blue with the rest of the text in black. It is this colour difference which makes the hyperlinks stand out. However, Nielsen [2] claimed that it was a bad decision to make hypertext links blue because only 2% of the cones on the retina are sensitive to blue making it a poor choice in terms of usability [3]. Nevertheless, Nielsen admits that the convention of the blue hyperlink should remain because users know that blue text denotes a hyperlink, making it easier for users to recognise hyperlinks more rapidly. This is supported by research on automatic attention which suggests that when a user consistently searches the same environment for the same information which is consistently represented in the same way, the processing becomes automatic [4,5]. This could also be true for hyperlinks because blue text in a webpage context almost always represents a hyperlink. Indeed, Campbell and Maglio [6] found that participants were quicker when searching for a target word in a webpage that was blue and underlined than target words that were black and underlined.

Very little research explores the impact of the saliency of words when reading for comprehension. Simola, Kuisma, Oorni, Uusitalo and Hyönä [7] explored reading in a Web environment and found that salient advertisements can distract attention and disrupt reading. If salient adverts can distract readers, it is conceivable that salient words may as well. White and Filik [8] examined bold words in passages of normal text. They found that bold text had shorter fixation durations suggesting that saliency in text can affect information processing and suggest their finding reflects the improved visual discriminability of the target words, making it easier to identify. There is also evidence suggesting that saliency can affect not just when we move our eyes, but also where we move them. Leyland, Kirkby, Juhasz, Pollatsek and Liversedge [9] examined eye movement behaviour during a reading experiment on fully or partially shaded words within the text and found that when a word was shaded it had an effect on saccadic targeting, influencing where the eyes move to. If only the first half of the word was shaded, the targeting was closer to the beginning of the word compared to when the entire word was shaded. Furthermore, partially shaded words were fixated for longer than fully shaded words, or non-shaded words, suggesting that visual non-uniformity (in that the shading was inconsistent with word boundaries) also affects when we move our eyes.

Recently, Gagl [10] asked participants to read text that featured target words that were either not highlighted or highlighted by being coloured in blue or by being underlined. Gagl found that highlighting a word by colouring it or underlining it had no negative or positive impact on reading during first pass. However, in total viewing times (which includes re-reading time of the word), there was an effect of whether the target was highlighted. The un-highlighted black words showed a reduced viewing time in comparison to the other conditions. This suggests that highlighting with colour or underlining increased re-reading. Gagl proposed that having hyperlinks coloured in blue is a good choice because it does not disrupt first pass reading, but attention is drawn to the highlighted words as is evident from re-reading so it serves the function of highlighting important information.

There has also been research into learning from electronic texts that suggest that hyperlinks do attract attention to them and that this attention actually assists in the retention of the hyperlinked word. The saliency of the hyperlinked words would ensure better acquisition and retention [11] and this idea is also compatible with the classic phenomenon called the Von Restorff effect [12], where items that ‘stand out’ are more likely to be remembered.

Turning to the linking function of hyperlinks, this information can be considered to be, in terms of cognitive processes, more high-level compared to the information that is exclusively contained in the lexical representation of the word that is hyperlinked. Hyperlinks denote a connection to other content somewhere else on the Web. Carr [13] suggested that hyperlinks within the text are a distraction and therefore hinder comprehension of the text. Having to evaluate hyperlinks and navigating a path through them is demanding and is an extraneous task to the act of reading itself. This means that having a hyperlink in the text that links to other content renders the act of reading more laborious and so from this perspective we expect this higher-level processing to be reflected in the eye movement measures during reading of the text.

In terms of a prediction for a high-level factor on reading, the current models of eye movements during reading do not make direct predictions for the impact of hyperlinks but the closest to a prediction that can be derived originates from the E-Z Reader model of eye movements during reading [14]. The EZ-Reader model suggests that higher-level processes intervene in eye movement control only when “something is wrong” and either send a signal to stop moving forward or to execute a regression. As a result, higher-level processes would exclusively impact the later eye movement measures (regressions and re-reading) so based on this model we hypothesise seeing effects of reading hyperlinks exclusively in the later eye movement measures.

Typographical cues have been shown to improve memory for the signalled content [15–18]. However, simply bolding or underlining the text does not automatically mean it will be remembered, the signal needs to be useful to the reader. Golding and Fowler [19] found that when the reader expected questions on specific details, underlining sections of text facilitated cued recall for those sections. The important information that the reader needed for the task was highlighted and this helped them find the information easily. However, when the reader was expected to provide an outline of text or a list of solutions to the problem discussed in the text, the readers did not experience any benefits from the signalling as it wasn’t useful for the task at hand. Thus, signals need to be relevant to the reader to assist them in their task.

There is also the issue that even if some signals are useful, will the addition of (even) more signals be more useful for the reader? If most of the text has some form of signal to cue the importance of the information, then the signal might not be as effective or as informative compared to when only the most important text is signalled. This “over-signalling” can reduce the effectiveness of typographical cues. For example, Lorch, Lorch and Klusewitz [20] asked individuals to read a four-page text after which they were tested on memory for specific target sentences. The text either contained no underlining (control), underlining of the target sentences (light signalling) or underlining of the target sentences and half of the non-target sentences (heavy signalling). Recall was improved when the text had light signalling, but performance was not different from the control condition when there was heavy signalling. If the signalling is not useful for the task, for instance when the signalling is seemingly meaningless, the reader will ignore it. Lorch, et al. [20] went on to replicate the control and light signalling conditions, but using capitalisation as the signalling tool instead of underlining. They found that reading was slower for the light signalling condition, but memory recall was improved. Upon further examination, they also observed that the readers slowed down on the signalled content alone and speeded up again when reading non-signalled content. This suggests that the reader may have thought the signalled content was important so decided to spend more time on it. During reading the reader needs to discriminate important and unimportant information and signals in the text can be used to assist the reader.

In terms of reading on the Web, hyperlinks could be said to be a typographical signal due to the fact that hyperlinks are a single word or short phrase that is salient from the rest of the text. Hyperlinked words could also be considered important by the reader and as such the presence of the hyperlink may add emphasis to that section of text.

The experiments here focus on how we read hyperlinked text and whether the links influence reading behaviour. In order to examine how links affect reading behaviour, we will first examine outside of a Web context any potential disruption of reading exclusively due to the target word being a salient colour compared to the rest of the text, before examining in a Web context whether this is due to the link being perceived as important due to the additional information that it can link to.

Three experiments were conducted to explore this issue. The first experiment, Experiment 1, explored whether a salient, coloured word negatively impacts reading behaviour outside of a hypertext context. Experiment 1 only used a single coloured word in a single-line sentence during reading for comprehension to explore the impact of saliency. The aim of Experiment 1 was to explore the impact of a single coloured word in a sentence and also to investigate if there was a difference between colours, or if simply being a salient word had an impact on reading. A follow-on experiment (Experiment 2), was conducted, exploring whether multiple coloured words had an impact on reading and we also include a word frequency manipulation to see if the difficulty of the word interacts with the fact that the word is coloured. A robust finding in eye movements during reading is that a high-frequency word receives shorter and fewer fixations than a low-frequency word [21,22]. This manipulation allowed us to examine whether there would be an additional cost of the colouring for words that are more difficult. Experiment 2 built upon Experiment 1 by exploring the impact of multiple coloured words in a sentence. By first exploring the impact of coloured words in text we can understand the impact of coloured words in plain text compared to coloured words shown in a hyperlinked environment, such as in Experiment 3. Experiment 3 explored whether perceiving the words as links influences reading behaviour by presenting the coloured words in text that can be perceived as hypertext. We also included a word frequency manipulation in this experiment in order to explore whether common lexical effects are present in hyperlinked text and to investigate if they are modulated by the word being hyperlinked. Together, these experiments assessed whether there is a difference between reading coloured words (embedded in words of a different colour) and reading hyperlinks and how this affects reading behaviour. In other words, Experiment 1 and 2 will help us to separate whether any observed effects seen in Experiment 3 are exclusively due to the saliency of a blue word or due to the fact that the blue words are hyperlinks in a hypertext environment.

As previously mentioned only 2% of the cones on the retina are sensitive to blue making it supposedly a poor choice in terms of usability [3], and this could impact reading behaviour as well if it is more difficult to read text in a certain colour. In line with previous research which has suggested that hyperlinks disrupt reading behaviour [2,13], we predicted that the coloured words would be fixated for longer because of the saliency of the coloured word. In Experiment 1, several colours were used for the target word to investigate whether blue was indeed particularly disruptive and we also predicted that specifically for grey target words that they would be fixated for longer due to their reduced contrast, thereby making them visually more difficult to process [23].

## Experiment 1

### Method

#### Participants

Thirty native English speakers (2 male, 28 female) with an average age of 19.80 years participated in exchange for course credits. All had normal or corrected-to-normal vision and no known reading disabilities.

#### Apparatus

Eye movements were measured with an SR-Research Eyelink 1000 eye tracker operating at 1000 Hz (1 sample every millisecond). Participants viewed the stimuli binocularly, but only the right eye was tracked. Words were presented in 14pt mono-spaced Courier font. The participant’s eye was 73 cm from the display; at this distance three characters equalled 1° of visual angle.

#### Materials and design

**Fig 1:**
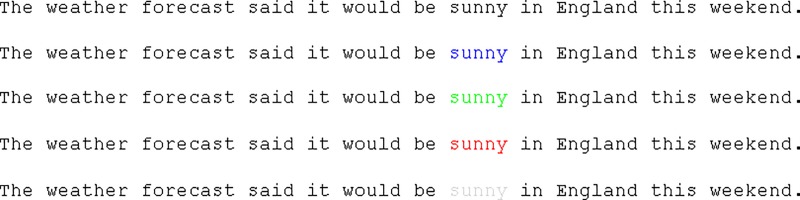
Example stimulus from Experiment 1 for the 5 different conditions.

Thirty sentences were used and a single target word in each sentence would appear in one of five colours, which correspond to the five experimental conditions (black (RGB: 0,0,0), blue (RGB: 0,0,255), green (RGB: 0,255,0), red (RGB: 255,0,0) or grey (RGB: 192,192,192); see Fig 1). The rest of the sentence was always rendered in black. A counterbalanced design was used in which each participant read one version of each of the thirty sentences with an equal number from each condition. Participants were instructed to read for comprehension and told that they would occasionally have to answer comprehension questions about the sentences. Comprehension questions were presented randomly in 25% of trials, they were simple yes/no questions and the accuracy of answering these was high (97.5% accuracy), indicating that participants were reading the text correctly.

#### Procedure

Before any of the experiments in this article took place, ethics approval was applied for, peer-reviewed and granted by the University of Southampton Psychology Department Ethics Committee. Ethics approval was sought and approved for all experiments within this article. Participants were given an information sheet and a verbal description of the experimental procedure and informed that they would be reading sentences on a monitor while their eyes were being tracked. They were told to read for comprehension and that they were to respond to comprehension questions presented after the sentences. If participants enquired about the colour of the words they were told the words were coloured at random and did not correspond to the comprehension questions. The participants were seated in front of the monitor and their heads were stabilised using a head and chin rest to reduce head movements. The initial calibration required approximately five minutes before the actual experiment began. At the beginning of each trial a fixation point was presented on the screen where the beginning of the text was set to appear. The participants were required to fixate this point before the sentence was presented to ensure that the first fixation fell on the first word of the sentence. When the participant had finished reading the text on the screen, they pressed a button to continue to the next trial. Each participant first read three practice trials to become familiar with the procedure. The experiment lasted approximately 15 minutes.

### Results

Trials where there was tracking loss were removed prior to the analyses. Fixations shorter than 80ms that were within one character of the previous or following fixation were merged and all remaining fixations shorter than 80ms or longer than 800ms were removed (resulting in the removal of 4.87% of the total dataset). Finally, when calculating the eye movement measures, data that were more than 2.5 standard deviations from the mean for a participant within a specific condition were removed (<1% of dataset). Data loss affected all conditions similarly.

Several eye-movement measures were calculated based on the target word. Skipping probability is the probability that a target word does not receive a direct fixation during the first-pass, first-fixation duration is the duration of the initial first-pass fixation on the target word, single fixation duration is the duration of the fixation if the reader made exactly one first-pass fixation on the target word, gaze duration is the sum of all first-pass fixations on the target word, go past time is the time between first fixating the word and moving past it to the right (including regressions that originate from the target word), and total time is the total amount of time spent on the target word during the whole trial, including any re-reading that might occur.

We ran Linear Mixed Models (LMMs) using the lme4 package (Version 1.1–12) [24] in R (Version 3.3.1) [25] to explore the impact of the colour of the target words on fixation times. Binominal models were used for the skipping probability measure.

The colour of the target word was included as a fixed factor, with treatment contrasts specifying black as the baseline in order to be able to compare reading of the target word embedded in a plain sentence without having a different colour with the reading of the target word rendered in another colour. Participants and items were included as random effects variables in a so-called maximal random model which included both intercepts and slopes for the colour factor [26]. If a model did not converge, the random effect structure was reduced first by removing the random effect correlations and then the interactions between the slopes and finally by successively removing the random effects explaining the least variance until the maximal converging model was identified. All the patterns observed in the models were identical whether they were run on log-transformed or untransformed fixation durations, allowing us to present the data run on the untransformed fixation durations in order to increase transparency. Absolute values of *t* equal to or bigger than 1.96 were interpreted a significant because for high degrees of freedom as is typically the case in LMMs, the *t* statistic approximates the *z* statistic.

The means for all of the eye movement measures for Experiment 1 are listed in Table 1. Participants were significantly less likely to skip a target word when it was not in black (see Table 2 for the LMM output). This suggests that the saliency of the coloured target word draws attention to it, making it more likely that participants will fixate on it.

**Table 1:** Means of eye movement measures for Experiment 1.

Target Word Colour	Skipping Probability (%)	First Fixation Duration (ms)	Single Fixation Duration (ms)	Gaze Duration (ms)	Go Past Time (ms)	Total Reading Time (ms)
Black	27 (19)	222 (36)	229 (54)	242 (47)	282 (106)	284 (66)
Blue	14 (15)	219 (49)	225 (80)	244 (55)	280 (95)	319 (87)
Green	11 (14)	234 (41)	243 (46)	260 (57)	339 (115)	364 (110)
Red	8 (10)	209 (36)	210 (44	229 (47)	279 (59)	305 (91)
Grey	13 (13)	244 (40)	247 (51)	279 (50)	337 (96)	354 (94)

Standard deviation in parentheses.

**Table 2:** Fixed effect estimates for all eye movement measures for Experiment 1.

	Skipping Probability			First Fixation Duration (ms)		
	Estimate	Std Error	*z* value	Estimate	Std Error	*t* value
Intercept	-1.28	0.30	-4.34 *	222.63	7.86	28.34 *
Blue	-0.97	0.35	-2.78 *	-4.22	9.30	-0.45
Green	-1.40	0.46	-3.03 *	10.82	8.38	1.29
Red	-1.53	0.37	-4.14 *	-13.98	8.30	-1.69
Grey	-0.98	0.39	-2.54 *	23.14	8.03	2.88 *
	Single Fixation Duration (ms)			Gaze Duration (ms)		
	Estimate	Std Error	*t* value	Estimate	Std Error	*t* value
Intercept	221.40	9.38	23.62 *	244.50	10.79	22.67 *
Blue	4.53	14.68	0.31	-3.56	11.15	-0.32
Green	14.67	9.86	1.49	15.07	11.08	1.36
Red	-8.15	10.95	-0.74	-16.91	11.01	-1.54
Grey	34.37	11.80	2.91 *	35.57	11.14	3.19 *
	Go Past Time (ms)			Total Reading Time (ms)		
	Estimate	Std Error	*t* value	Estimate	Std Error	*t* value
Intercept	279.04	19.11	14.6 *	282.65	18.55	15.24 *
Blue	0.22	24.90	0.01	32.52	17.74	1.83
Green	57.83	26.56	2.18 *	76.59	17.64	4.34 *
Red	-3.18	23.73	-0.13	19.91	17.52	1.14
Grey	57.75	20.69	2.79 *	73.03	17.74	4.12 *

*z or *t*>|1.96|

However, there were no statistically significant differences in any of the fixation time measures across the conditions except when the target word was grey. The reduced contrast of the target word in this condition increased the fixation time on that word both in early and late eye movement measures compared to any other condition because it was more difficult to visually process (e.g., [23]). Also, there was a significant difference in the later eye movement measures for when the target word was shown in green: Participants spent longer on the green target word in total reading time and were more likely to regress back to re-read the preceding text, as shown by the increased go past times. This suggests that the participants also found the green word a bit more difficult to process and we suggest this is also due to the reduced contrast of the green text compared to the other colours used besides grey (see Fig 1). To verify this, we determined the luminance of the colours used on the screen during the experiment. Luminance is measured in candela per square metre (cd/m2). If we look at the luminance of each of the colours used we notice that the grey and green are similar in luminance (grey:80.0 cd/m2; green: 73.2 cd/m2) and closer to the luminance of the white background (103.0 cd/m2) than any of the other colours; blue (10.2 cd/m2), red (18.6 cd/m2) and black (0.7 cd/m2). However, it is not clear why this effect of luminance would manifest itself exclusively in later eye movement measures for the green colour.

### Discussion

Experiment 1 demonstrated that a coloured word is less likely to be skipped, perhaps because the reader thought the colour serves as a signal that the word might be important in some way [19,20]. Or it could simply be because the coloured word was salient against the rest of the text and attracted the readers’ eye [7,9,27]. There was no negative impact on reading behaviour in terms of fixation times when a word was coloured unless when the colour was associated with reduced contrast making it more difficult to read as seen when the target word was grey or green.

## Experiment 2

Experiment 2 follows on from Experiment 1 by including multiple coloured words in a sentence to investigate if additional salient words will have an impact on reading behaviour compared to the single coloured word presented in Experiment 1. If a single coloured word causes a reduction in word skipping for that word, will it occur for all salient words in a sentence when there are multiple? Or will an effect of “over-signalling” occur where the signal of importance is reduced when words are coloured seemingly randomly [19,20]. Note that on the Web the presence of multiple hyperlinks across the screen will likely be the default as opposed to a single coloured word.

In Experiment 2 we only use the colour blue or black for our target words to feature in our coloured word condition. We choose to only use blue or black to represent the colours most often used in a Web environment, where black text tends to represent the unlinked text and the blue text represents the hyperlinked text. We also include a word frequency manipulation to explore whether word difficulty interacts with whether a word is presented in a salient colour (compared to the rest of the text) or not. A reader typically spends more time fixating a difficult or low frequency word than an easy or high frequency word [21,28,29]. We want to explore if a reader will spend even longer processing a more difficult word if it is also coloured/salient.

### Method

#### Participants

36 native English speakers (17 male, 19 female) with an average age of 25.25 years participated in exchange for course credits. All had normal or corrected-to-normal vision and no known reading disabilities. These participants did not take part in either Experiment 1 or 3.

#### Apparatus

The apparatus was identical to the one used in Experiment 1.

#### Materials and design

Seventy-two sentences were used and there were six conditions with each participant seeing twelve sentences in each condition according to a Latin square design. Each sentence contained a target word of which we manipulated the word frequency and how many coloured words were present in the sentence, being either no coloured words, one word (the target word) or three words (the target plus two other words chosen randomly). For the word frequency manipulation where the target word is either high or low frequent the word length was matched for each pair of stimuli and was either 4 or 5 characters in length (on average 4.63 characters). The word frequencies were taken from the Hyperspace Analogue to Language (HAL) corpus [30], which consists of approximately 131 million words gathered across 3,000 Usenet newsgroups. The frequency norms were used to extract both high and low frequency words to create the experimental stimuli. The high frequency words had an average log transformed HAL frequency of 10.16 and the low frequency words has an average log transformed HAL frequency of 6.39 according to the norms collected in the HAL corpus. There was a significant difference between the word frequency for the low and high frequency stimuli *t*(71) = 28.93, *p*<0.001.

**Fig 2:**
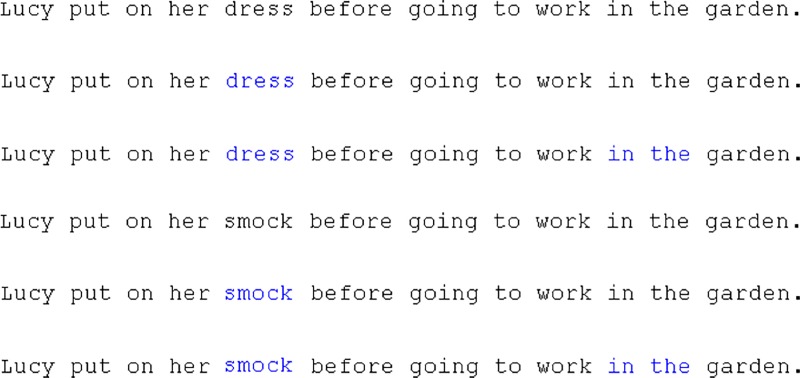
Example stimulus from Experiment 2 of the six different versions of a single stimuli.

In total, there were six conditions (see Fig 2). Participants were instructed to read for comprehension and told that they would occasionally have to answer comprehension questions about the sentences. Comprehension questions were presented after 25% of trials and the accuracy of answering these was high (95.50% accuracy), indicating that participants were reading the text for comprehension.

The first three represent the high frequency conditions with the target word “dress” and it is displayed with either no, one or three coloured words. The last three sentences represent the low frequency conditions with the target word “smock”.

#### Procedure

Participants were given an information sheet and a verbal description of the experimental procedure and informed that they would be reading sentences on a monitor while their eyes were being tracked. They were told to read for comprehension and that they were to respond to comprehension questions presented after the sentences. If participants enquired about the colour of the words they were told the words were coloured at random and did not correspond to the comprehension questions. The procedure was identical to Experiment 1. The experiment lasted approximately 30 minutes.

### Results

The data cleaning procedure and eye movement measures used were identical to those used in Experiment 1 and resulted in the removal of 4.46% of the total dataset. Finally, when calculating the eye movement measures, data that were more than 2.5 standard deviations from the mean for a participant within a specific condition were removed (<1% of dataset). Data loss affected all conditions similarly.

We ran LMMs using the lme4 package (Version 1.1–12) [24] in R (Version 3.3.1) [25] to explore the impact of word frequency and of the number of salient coloured words in the sentence on fixation times. Binominal models were used for the skipping probability measure. Word frequency and number of coloured words were included as fixed factors, with high frequency target word and having no coloured words present as the baseline in order to be able to compare reading a plain sentence with no salient target words to a sentence with salient words and/or a sentence with a low frequency target word. Participants and items were included as random effects variables. A maximal random model was initially specified for the random factors [26]. If a model did not converge, it was pruned by first removing the random effect correlations, then the interactions between slopes and finally by successively removing the random effects explaining the least variance until the maximal converging model was identified. Model comparisons were also utilised to compare models for the best fitting model for the data for each measure. For most of the measures the best fitting model required the removal of the interaction between word frequency and the number of coloured words. The only exception is the go past time measure where including the interaction gave a better fit for the data. For word skipping the participants and items intercepts were allowed to vary. For the measures of first fixation durations, single fixation durations, gaze durations and total time the intercept for the items variable was allowed to vary, whilst the participant variable included both the intercept and the slope obtained for the additive effects of word frequency and number of coloured words. Finally, for go past times the items variable intercept was allowed to vary, whilst the participant variable included both the intercept and the slope obtained for the interaction between word frequency and number of coloured words, but no correlations between random effects was included. All the patterns observed in the models were identical whether they were run on log-transformed or untransformed fixation durations, allowing us to present the data run on the untransformed fixation durations in order to increase transparency.

The means for all of the eye movement measures for Experiment 2 are listed in Table 3. Afterwards, an additional contrast was run directly comparing one coloured word to three coloured words to explore whether more links effects reading behaviour (see Table 4 for the LMM statistics). We observe a significant effect in skipping probability and fixation durations for the target word frequency where the low frequency word is less likely to be skipped and has longer fixation durations when it is fixated, replicating previous research [21,28]. The frequency effect does not interact with whether the target word was coloured or not. Indeed, for most measures the interaction did not contribute to the fit of the model and was removed. We replicate the reduction in skipping observed in Experiment 1, with a significant difference between whether the target word was coloured or not and less skipping when there was a single coloured word (see Table 4). However, this effect is only present when there is only a single coloured word. When there are three coloured words the reduction in skipping does not happen and the skipping rates are in line with reading when there are no coloured words in the text. In terms of fixation durations, there was no effect of the number of coloured words in the sentences on fixation duration measures (except in total time) suggesting salient/coloured words have very little impact on reading behaviour. There is a main effect of number of coloured words in total time spent on the target word where significantly more time (38 ms on average) is spent on the target words when there is only one coloured word present in the sentence. This suggests that the readers are going back and rereading the single coloured word as we do not see this effect in any of the first-pass reading measures.

**Table 3:** Means of eye movement measures for Experiment 2.

Word Frequency	Number of Coloured Words	Skipping Probability (%)	First Fixation Duration (ms)	Single Fixation Duration (ms)	Gaze Duration (ms)	Go Past Time (ms)	Total Reading Time (ms)
High	0	21 (15)	207 (27)	210 (27)	223 (33)	256 (56)	264 (63)
High	1	17 (14)	203 (30)	202 (36)	215 (37)	253 (82)	268 (72)
High	3	19 (14)	202 (27)	205 (35)	217 (37)	255 (83)	267 (78)
Low	0	15 (12)	219 (29)	229 (40)	245 (41)	282 (69)	293 (74)
Low	1	13 (12)	217 (31)	223 (36)	243 (47)	303 (81)	315 (99)
Low	3	14 (15)	214 (34)	221 (37)	238 (49)	292 (101)	296 (92)

Standard deviation in parentheses.

**Table 4:** Fixed effect estimates for all eye movement measures for Experiment 2.

	Skipping Probability			First Fixation Duration (ms)		
	Estimate	Std error	*z* value	Estimate	Std error	*t* value
Intercept	-1.51	0.15	-9.91 *	206.47	3.96	52.13 *
Frequency	-0.40	0.10	-3.79 *	12.96	3.18	4.07 *
No of Links 1	-0.25	0.13	-1.97 *	-2.85	3.75	-0.76
No of Links 3	-0.16	0.13	-1.30	-4.57	3.41	-1.34
Frequency * No of Links 1						
Frequency * No of Links 3						
Contrast–No of links 1 vs No of Links 3	0.09	0.13	0.67	-1.72	3.22	-0.53
	Single Fixation Duration (ms)			Gaze Duration (ms)		
	Estimate	Std error	*t* value	Estimate	Std error	*t* value
Intercept	210.22	4.62	45.55 *	222.56	5.21	42.7 *
Frequency	17.43	3.64	4.78 *	23.71	4.76	4.99 *
No of Links 1	-5.16	4.61	-1.12	-5.28	4.46	-1.18
No of Links 3	-6.44	3.97	-1.63	-6.86	4.53	-1.51
Frequency * No of Links 1						
Frequency * No of Links 3						
Contrast–No of links 1 vs No of Links 3	-1.29	4.04	-0.32	-1.57	4.08	-0.39
	Go Past Time (ms)			Total Reading Time (ms)		
	Estimate	Std error	*t* value	Estimate	Std error	*t* value
Intercept	258.73	9.91	26.10 *	260.57	10.73	24.28 *
Frequency	23.89	10.68	2.24 *	37.32	7.50	4.976 *
No of Links 1	-5.74	12.42	-0.46	13.60	6.80	2.001 *
No of Links 3	-3.58	11.24	-0.32	3.06	6.82	0.45
Frequency * No of Links 1	28.27	17.36	1.63			
Frequency * No of Links 3	12.93	14.55	0.89			
Contrast–No of links 1 vs No of Links 3	2.16	11.65	0.19	-10.54	6.74	-1.56

*z>|1.96| *t>|1.96|

### Discussion

Experiment 1 demonstrated that a single coloured target word is less likely to be skipped. Experiment 2 replicates this finding but shows that when there are multiple coloured words we do not observe the same reduction in skipping. In Experiment 1 and 2, we thus observe a reduction in skipping but only from a single coloured target word. This is presumably because a single coloured word in a sentence works as a signal of importance to that particular word [20]. When there are multiple words being highlighted in the sentence this results in “over-signalling” which can reduce the effectiveness of typographical cues. The reader does not perceive the signal of the coloured text as important if it is not useful to them. When one word is coloured it might suggest that that particular word is important in the sentence. However, when three words in the sentence are coloured at random the signal seems meaningless and serves no use to the reader so is therefore ignored.

An alternative, not mutually exclusive, explanation is that by including multiple coloured words we are reducing the saliency of the coloured target word [1]. A single target word is presumably very salient in a single line of text, but less salient when there are other coloured words in that same line of text. Therefore, when the saliency is reduced due to the presence of multiple coloured words in Experiment 2, we no longer observe the reduction in skipping of a single coloured target word observed in Experiment 1.

## Experiment 3

Experiment 3 explored whether perceiving the coloured words as hyperlinks influences reading behaviour and whether they are processed differently to coloured words in plain text. Previously it has been suggested that the hyperlinked words would be fixated for longer due to the reduced visual discriminability of the blue words [2,13]. However, because blue hyperlinks are so commonplace in webpages, the processing may become automatic [4,31]. This could mean that in the case of hyperlinks, blue text is automatically processed as being a hyperlink without an additional cost because blue text tends to always be a hyperlink. We implemented a word frequency manipulation of the hyperlinked words in order to explore whether common lexical effects are present in hyperlinked text and to investigate if they are modulated by the word being hyperlinked. For word skipping, we predict that high frequency words are skipped more often than low frequency words [21]. Whereas Experiment 2 has shown that when multiple words are coloured there is no effect on word skipping of the colouring (compared to reduced skipping when only one word is coloured), the signal function of colouring which indicates the presence of a hyperlink might draw attention to the word and as such increase the chance of fixating the hyperlinked word, reducing skipping for these words.

Hyperlinks indicate that the word links to other content, a signal which can be considered from a cognitive perspective to be more high-level compared to the information exclusively retained in the lexical representation of the word. The prediction for how this additional high-level information will affect eye movement behaviour comes from the E-Z Reader model of eye movement control [14]. This model suggests that higher-level processes intervene in eye movement control only when “something is wrong” and either send a signal to stop moving forward or a signal to execute a regression. This is exclusively seen to impact the later eye movement measures so we expect to see hyperlinks impacting fixation durations in the later eye movement measures even though the signal that there is a link present in a word does not necessarily means “something is wrong”. Note that this model was not specifically designed to account of effects of hyperlinking a word, but does make global predictions for any higher level influence on eye movements during reading.

Experiments 1 and 2 explored the impact of colour/saliency to see if simply colouring a single word or multiple words has an impact on reading behaviour, because hyperlinks are of course firstly coloured/salient words in the text on webpages. However, hyperlinks also represent much more, they are the links to other webpages and are used as a form of navigation. In Experiment 3, we move forward from Experiment 1 and 2 in that we create trials that resemble a real Web environment, but we do not (yet) allow the readers to navigate (i.e. click on the links), only to read the content presented to them. This will help us tease apart the impact of the visual experience of reading hypertext from the additional impact of navigating a Web environment.

### Method

#### Participants

Thirty-two native English speakers (3 male, 29 female) with an average age of 19.72 years participated in exchange for course credits. All had normal or corrected-to-normal vision and no known reading disabilities. All these participants also took part in Experiment 1 first.

#### Apparatus

Identical apparatus to Experiment 1 and 2.

#### Materials and design

The stimuli in Experiment 3 consisted of twenty edited Wikipedia articles (example stimuli available: https://goo.gl/JLvvMD). Eighty target words were embedded in carrier sentences (one target word per sentence) and four carrier sentences were inserted into each Wikipedia article. The font was a mono-spaced font and the line spacing was approximately three-line spacing to improve the discrimination of fixations between lines. Although we altered the text format to improve discrimination between lines, the test sufficiently carried the impression of being a Wikipedia page by including the title banner and sidebar. The text was created by taking existing Wikipedia articles on neutral topics, and inserting four experimental sentences into the existing text. The experimental sentences fitted in with the text already present and contained a target word. The rest of the text on each screen was identical to the source material on Wikipedia, including which words were linked. This decision was made so that the articles were as close to a natural Web environment as possible. The Wikipedia articles were ten to twelve lines long. The target words were nouns and the location of the target words was scattered across the sentences, but they were never on the start or end of a line. The target words within these articles were either displayed in blue or black to denote if the word was a hyperlink or not, respectively. There was also a word frequency manipulation where the target word is either high or low frequent. So in total there were 4 conditions in a 2 x 2 within participants design The word frequencies were taken from the Hyperspace Analogue to Language (HAL) corpus [30], and these norms were used to select both high and low frequency words to create the experimental stimuli. The high frequency words had an average log transformed HAL frequency of 9.91 and the low frequency words has an average log transformed HAL frequency of 5.75. There was a significant difference between the high and low word frequency stimuli, t(79) = 24.61, p<0.001. The target words were matched on word length and were between 4 and 7 characters in length (5.24 characters on average). Participants were instructed to read for comprehension and told that they would be have to answer comprehension questions about the sentences, and these appeared after all trials.

#### Procedure

Participants were given an information sheet and a verbal description of the experimental procedure and informed that they would be reading passages on a monitor while their eyes were being tracked. They were told to read for comprehension and that they were to respond to comprehension questions presented after the sentences. The comprehension questions were simple and required a yes or no response. The comprehension questions were present to ensure the participants were reading the text displayed to them for comprehension. The total accuracy for the comprehension questions was 95.31%. The participants were seated in front of the monitor and their heads were stabilised using a head and chin rest to reduce head movements and ensure reliable eye tracking data. The initial calibration required approximately five minutes before the actual experiment began. At the beginning of each trial a fixation point was presented on the screen where the beginning of the text was set to appear. The participants were required to fixate this point before the trial began to ensure that the first fixation fell on the first word of the sentence. When the participant had finished reading the text on the screen, they pressed a button to move onto the next trial. Each participant first read two practice trials to become familiar with the procedure. Comprehension questions were presented on every trial to ensure each article was read in full and the experiment lasted approximately 45 minutes.

### Results

The data cleaning procedure and eye movement measures used were identical to that used in Experiment 1 and 2 (resulting in the removal of 4.47% of the total dataset). Finally, when calculating the eye movement measures, data that were more than 2.5 standard deviations from the mean for a participant within a specific condition were removed (<1% of dataset). Data loss affected all conditions similarly.

We ran LMMs using the lme4 package (Version 1.1–12) [24] in R (Version 3.3.1) [25] to explore the impact of word frequency and the target word being displayed as a linked or unlinked word (binominal models were used for the skipping probability measure). Word Frequency and Word Type (whether the word was linked or unlinked) were included as fixed factors. Participants and items were included as random effects. A maximal random model was initially specified for the random factors [26]. If a model did not converge, it was reduced by first removing the random effect correlations, the interactions between the slopes and finally by successively removing the random effects explaining the least variance until the maximal converging model was identified. For most of the measures the intercept for the items variable was allowed to vary and the participant variable included both the intercept and the slope obtained for the interactive effects of Word Frequency and Word Type. The only exceptions were skipping proportion and gaze duration where the intercept for the items variable was allowed to vary and the participant variable included both the intercept and the slope for the effect of Word Frequency. The effect of Word Type had to be removed to allow the models to converge.

All the patterns observed in the models were qualitatively identical whether they were run on log-transformed or untransformed fixation durations, allowing us to present the data run on the untransformed fixation durations in order to increase transparency. Successive differences contrasts were used such that the intercept corresponds to the grand mean and the fixed effects of Word Frequency and Word Type to main effects. The means for all the eye movement measures for Experiment 3 are listed in Table 5 and the LMM statistics in Table 6.

**Table 5:** Means of eye movement measures for Experiment 3.

Word Frequency/Word Type	Skipping Probability (%)	First Fixation Duration (ms)	Single Fixation Duration (ms)	Gaze Duration (ms)	Go Past Time (ms)	Total Reading Time (ms)
High/Linked	43 (23)	216 (34)	214 (35)	227 (36)	298 (123)	261 (54)
High/Unlinked	46 (24)	215 (32)	219 (40)	228 (38)	291 (84)	266 (67)
Low/Linked	40 (24)	232 (45)	249 (47)	258 (47)	364 (112)	320 (68)
Low/Unlinked	43 (24)	231 (38)	238 (44)	251 (47)	306 (88)	297 (60)

Standard deviation in parentheses.

**Table 6:** Fixed effect estimates for all eye movement measures for Experiment 3.

	Skipping Probability			First Fixation Duration (ms)		
	Estimate	Std error	*z* value	Estimate	Std error	*t* value
Intercept	-0.33	0.22	-1.52	222.98	5.46	40.88 *
Word Frequency	-0.15	0.09	-1.67	18.71	5.12	3.65 *
Word Type	-0.12	0.09	-1.36	1.18	5.12	0.23
Word Frequency * Word Type	0.03	0.18	0.16	4.20	8.48	0.50
	Single Fixation Duration (ms)			Gaze Duration (ms)		
	Estimate	Std error	*t* value	Estimate	Std error	*t* value
Intercept	229.36	6.16	37.23 *	239.74	6.67	35.95 *
Word Frequency	28.77	5.99	4.8 *	32.45	4.49	7.22 *
Word Type	3.55	4.99	0.71	1.75	5.53	0.32
Word Frequency * Word Type	14.02	8.47	1.66	13.50	8.97	1.51
	Go Past Time (ms)			Total Reading Time (ms)		
	Estimate	Std error	*t* value	Estimate	Std error	*t* value
Intercept	311.22	12.93	24.06 *	286.58	9.96	28.78 *
Word Frequency	40.25	14.92	2.70 *	46.06	9.11	5.06 *
Word Type	20.14	14.77	1.36	7.31	8.67	0.84
Word Frequency * Word Type	49.47	23.31	2.12 *	31.94	14.03	2.28 *

*z>|1.96|

**Fig 3:**
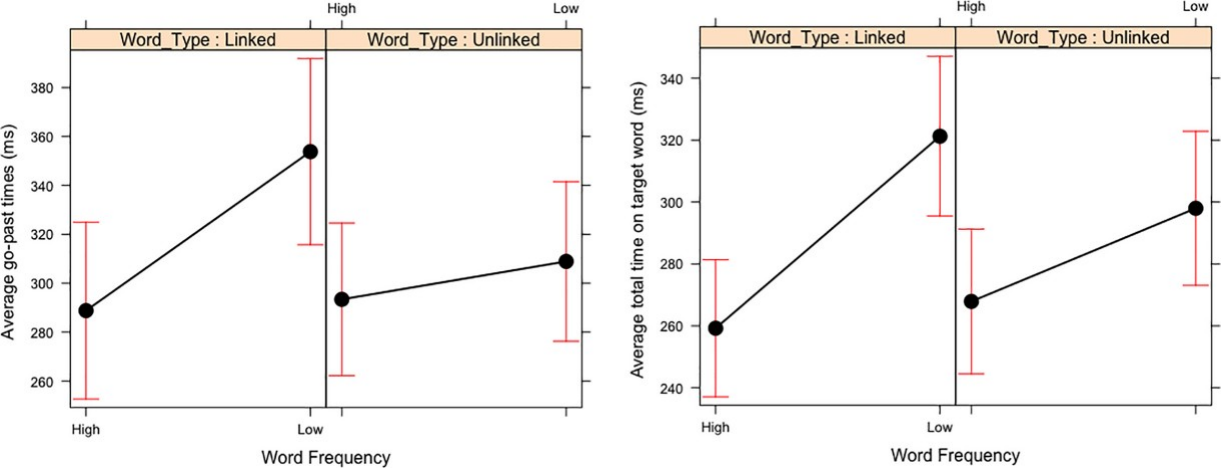
Two-way interaction between word frequency x word type interaction in Experiment 3. Means and 95% Confidence Intervals for go-past times and total reading times.

The effect of Word Frequency was present in all fixation-based eye movement measures. However, for word skipping, the high frequency words were numerically skipped more often than the low frequency words but this effect did not reach statistical significance. The high frequency words had significantly shorter fixation times than the low frequency word when they were fixated. This replicates previous experiments that have demonstrated that low frequency words are skipped less often and have longer fixations times because they are more difficult to process than highly frequent words [21]. However, in go-past times and total time the effect of Word Frequency was qualified by an interaction with Word Type (Fig 3) which we will discuss in detail below.

Replicating the results of Experiment 1 and 2 for coloured words, there were no significant differences between whether the target word was linked or not in the early fixation duration measures (first fixation durations, single fixation durations and gaze durations). This suggests that having a word linked does not make it any more difficult to process in first-pass reading. However, there was also no difference in the amount of skipping observed in Experiment 3 between when the target word was linked or not. This is especially interesting because in Experiment 1 and Experiment 2 when a single word was coloured it was less likely to be skipped. However, in Experiment 2 when there was more than one word coloured in a sentence, the reduction in skipping observed for single coloured words was not observed. Therefore Experiment 3 replicates the findings of Experiment 2 with no reduced skipping for coloured words when there are multiple words. Moreover, it shows that the fact that the coloured words are hyperlinks does not influence skipping rates.

In the later eye movement measures of go-past times and total reading times we observed a significant interaction between Word Frequency and Word Type. As Fig 3 shows, the low frequency, linked words had significantly longer go-past times and total reading times on the target word compared to the other three conditions. This interaction was only present in the late eye movement measures which suggest that the low frequency, linked word causes regressive eye movements due to difficulty in processing, in other words the participants are reading the low frequency linked words and rereading the preceding content to re-evaluate it.

### Discussion

Experiment 3 demonstrated that coloured words in passages of text in an environment resembling the Web do not have a negative effect on the early measurements of reading behaviour. In Experiment 3, we observed a main effect of word frequency where low frequency words receive longer fixation times than high frequency words. Importantly, this frequency effect was also qualified by an interaction with whether the target word was hyperlinked or not in the later eye movement measures. There were significantly longer eye movement measures in go-past times and total reading times but only when the target word was hyperlinked and low frequent (see Fig 3). This finding is compatible with the E-Z Reader model of eye movement control [14] that suggests that high-level processes only intervene in eye movement control when the processing is difficult and this is exclusively observed in the later eye movement measures, as observed in Experiment 3. This might also explain why there are no differences in the fixation times when the target word is hyperlinked and high frequent, only when the target word was hyperlinked and the word itself difficult (i.e. low frequency) was the processing difficulty sizeable enough to trigger re-reading.

## General discussion

Combined these three experiments allowed us to examine the potential differences between reading text with and without a coloured word embedded in the sentence and when this coloured word was a hyperlink or not.

Experiment 1 demonstrated that a coloured word is less likely to be skipped when it is the only coloured word in the sentence, but that making a word coloured does not negatively impact reading behaviour as measured in fixation durations unless the colour has reduced contrast making it difficult to read, as was seen when the target word was grey or green.

Experiment 2 replicated Experiment 1 and showed a reduction in skipping of the target word when it was coloured. However, this reduction of skipping rates was not observed when there were multiple coloured words within the sentences displayed. There was also a standard frequency effect showing less skipping of and longer fixations on low frequency words [21,28], and frequency did not interact with whether the word was coloured. There are two possible suggestions to why there was a reduction in word skipping for a single coloured word, but not when there were multiple coloured words in the text. One suggestion is there could be an effect of over-signalling, where the importance of the coloured words gets reduced when there are too many words coloured randomly and the reader could not utilise the signal as it was meaningless to them [19,20]. The alternative suggestion is that the saliency of the target word is reduced when there are multiple coloured items present in the sentence [1]. A single target word is presumably very salient in a single line of text, but less salient when there are other coloured words in that same line of text.

Experiment 3 demonstrated that there is a difference between processing a word that is coloured during reading and a hyperlinked word. We observed a significant difference in go-past times and total reading times between whether the target word was hyperlinked or not in the reading of the Wikipedia pages, and this effect was qualified by an interaction with frequency. The hyperlinked, low frequency words had longer fixation times in these measures which indicated that the reader had difficulty integrating and processing the low frequency word when it was hyperlinked. As a result, participants were more likely to reread the preceding content to re-evaluate it. A hyperlink on a word indicates that that word is important and implies there is more information behind the hyperlink regarding the word or topic that the hyperlinked word corresponds to. When the hyperlinked word is a low frequency word the reader may wonder why that word is hyperlinked and want to re-evaluate the preceding content presumably to make sure that they understood it, or try to decide why it is important.

The longer reading time in the later eye movement measures on low frequency, hyperlinked words can also be discussed from a signalling perspective. We found in Experiment 2 that if most of the text has some form of signal to cue the importance of the information, then the signal might not be as effective or as informative compared to when only the most important text is signalled [20]. The signal needs to be useful to the reader in order for them to try and utilise it [19]. In Experiment 2 the signalling served no purpose, but in a hypertext-like environment, like in Experiment 3, it could serve a purpose. For example, the hyperlinked words could be suggesting that those pieces of text are important parts of the text and the reader may want to pay attention to them. When the target word was low frequency and hyperlinked they may have wondered why that particular word was linked and want to reassess the information preceding it to evaluate it.

In Experiment 1 a single coloured word was significantly less likely to be skipped than black words, and this is replicated in Experiment 2 where there are single coloured words in the text. However, in Experiment 2 when there were multiple coloured words in the text, there was no similar reduction in skipping rates. Similarly, in Experiment 3 there were also no differences in skipping rates when the word was hyperlinked/coloured versus when it was unlinked. This suggests that the reduced skipping of the coloured target words observed in Experiment 1 is likely restricted to when there is only one word that is coloured. When multiple words are coloured (even when this is in other trials but in the same experiment) the coloured word will presumably be perceived as less attention grabbing. Moreover, in Experiment 3 the colouring did serve a purpose (it indicated a hyperlink) although our current design does not allow is to tease apart whether the absence of a decreased skipping of coloured words in Experiment 3 compared to 1 is due to the presence of multiple coloured words reducing the coloured words saliency and/or because the colouring was meaningful as a signal.

Note that we are of course fully aware that there are more differences between Experiment 2 and Experiment 3 than whether the sentence is presented in a Web environment or not (e.g. Experiment 2 presents single sentences whereas Experiment 3 presents paragraphs). However, we believe Experiment 2 allows us to rule out the attention-grabbing effect of a coloured word when more than 1 word is present regardless of the presentation format. Radach, Huestegge, and Reilly [32] found that total viewing times for words were greater for passages of text, however, first pass viewing times were shorter compared to reading a sentence (see also [33]). This suggests that when the text is displayed as a passage the reader will make a quick first pass over the text and then engage in re-reading, a behavioural pattern not observed or at least not to the same extent when the participants read the text as single lines. Note however, that this difference between sentence and paragraph reading might impact some of our findings, such as the frequency effect not reaching statistical significance for word skipping in Experiment 3 as Radach et al. [32] observed reduced frequency effects in paragraph reading compared to single sentence reading. However, to examine the influence of hyperlinks it makes sense to study them in a paragraph setting as single sentences will be relatively rare in a Web environment.

The present set of experiments represent the first steps in understanding how we read hyperlinked text using eye-tracking to examine the reading behaviour. A hyperlink is not just a salient word in a passage of text, it also denotes that there may be more information behind that hyperlink. Enriching hypertext documents with large numbers of links that are automatically generated (as seen in [34]) may cause disruption to reading behaviour. Hyperlinks do not necessarily cause a disruption to reading, but our research here has shown that if you hyperlink a low frequent/difficult word when there is not a strong reason to do so there is re-reading of the content to assess why that particular word is hyperlinked.

These experiments have shown that coloured text in itself does not hinder reading, but also that coloured hyperlinks can cause the participant to reread previous content if the word is a low frequent/difficult word in order to re-evaluate the content. Although the participants could not click and navigate the hyperlinks, this experiment at least shows that having hyperlinks shown as salient blue text does not negatively impact on reading behaving. It does increase re-reading when a reader reaches a low frequency, hyperlinked word, but this is not necessarily a negative behaviour as it can fulfil a necessary function during the processing of hyperlinked content on the Web.

However, the hyperlinks do signal that the hyperlinked words are special/important in some way. Our current findings were first reported in a conference proceeding by Fitzsimmons, Weal, and Drieghe [35] where a pilot study preceding the current experiments was presented. Gagl [10] partly replicates our current finding in his paper. He examined single lines of text in a non-Web environment with a target word either displayed in black or blue and either underlined or normal. Gagl used an invisible boundary paradigm (comparing fixation times after a degraded vs normal preview) to investigate if there is a perceptual disadvantage of hyperlinks (blue target words). There was no frequency manipulation, but the target words had a similar frequency to our low frequency words. Gagl found no preview effect of colour. However, in re-reading measures, there were increased fixation times for blue words compared to black words. This replicates our finding in Experiment 3 suggesting there is no detectable perceptual disadvantage of coloured words, but increased attraction of attention resources, after first pass reading.

In terms of Web design and layouts, the present results highlight the importance of carefully considering which words are hyperlinked in webpages. The key lesson for Web designers that we have found here is that coloured words do not have any negative impact on reading behaviour. This is the case no matter the colour, unless the contrast between the text colour and the background colour is low, as seen in the longer fixation times on the low-contrast grey/green words in Experiment 1. Therefore, efforts made in Web development to avoid using blue as the hyperlink colour and instead using a different colour may have no positive influence for the reader reading the text, but instead make it more difficult for the reader to know what is a hyperlink when they are expecting it to conform to the convention of hyperlinks being denoted in blue. Additionally, we showed more re-reading for the low-frequency, hyperlinked words which suggests that Web developers should consider how often they want to use low-frequency hyperlinked words if the importance of having these links present is not crucial as they may wish to avoid excessive re-reading of the content. It is difficult to tell if our finding is due to the reader re-reading because they are confused as to why the low-frequency word is hyperlinked or another reason. Further research is needed to explore this issue.

Even though in the current experiments participants only engaged in reading behaviour and did not have to make decisions and click any hyperlinks, there was still a significant difference between reading a coloured word and a coloured word in a Web-like environment. The suggestion of a blue word representing a hyperlink is enough, in a Wikipedia/Web environment, to influence eye movement behaviour in all likelihood associated with the processing of a hyperlink even without the ability to click the hyperlinks.
